# Biliothorax: A Rare Case of Dyspnea

**DOI:** 10.7759/cureus.55838

**Published:** 2024-03-09

**Authors:** Rodrigo Rufino, Daniela Rodrigues, Marta Carinhas, Vera Luís, António Cardoso

**Affiliations:** 1 Internal Medicine, Centro Hospitalar Barreiro-Montijo, Barreiro, PRT

**Keywords:** emergency department, respiratory failure, pulmonary adenocarcinoma, bilious pleural effusion, biliothorax

## Abstract

Biliothorax is the presence of bile in the pleural cavity. This condition is rare, and it usually results as a complication of hepatobiliary procedures. The authors present a case of an 87-year-old female who was admitted to the emergency department with the acute onset of severe dyspnea. A chest X-ray and CT revealed a large right-lung pleural effusion that, after thoracentesis, confirmed the presence of biliothorax. It is important to consider this entity when confronted with an effusion liquid of a dark greenish color, as a delay in diagnosis and management may be life-threatening.

## Introduction

Biliothorax is an extremely rare clinical condition characterized by the presence of bile in the pleural cavity [1-3]. Most cases are unilateral (right-sided) because of the proximity to the liver and biliary system, with a bilateral presentation being extremely rare [3]. The main cause is usually related to complications of hepatobiliary procedures [1,4-7]. Some of these complications include malignant or benign biliary obstruction, fistulas after hepatobiliary invasive procedures, and abscesses within the liver or subdiaphragmatic region [2]. The diagnosis of bilious pleural effusion can be made using the ratio of pleural fluid total bilirubin to serum total bilirubin and the presence of pleural glycolic acid [8].

We report the case of an 87-year-old woman who presented to the emergency department (ED) with an acute onset of severe dyspnea. A chest X-ray and CT revealed an extensive right pleural effusion. A diagnostic thoracocentesis revealed a dark greenish fluid, suggestive of biliothorax.

## Case presentation

An 87-year-old autonomous woman with a history of pulmonary adenocarcinoma of the right upper lobe with pleural and bone metastasis, with a need for recurrent bloody pleural drainage, was admitted to the ED because of the acute onset of severe dyspnea.

**Figure 1:**
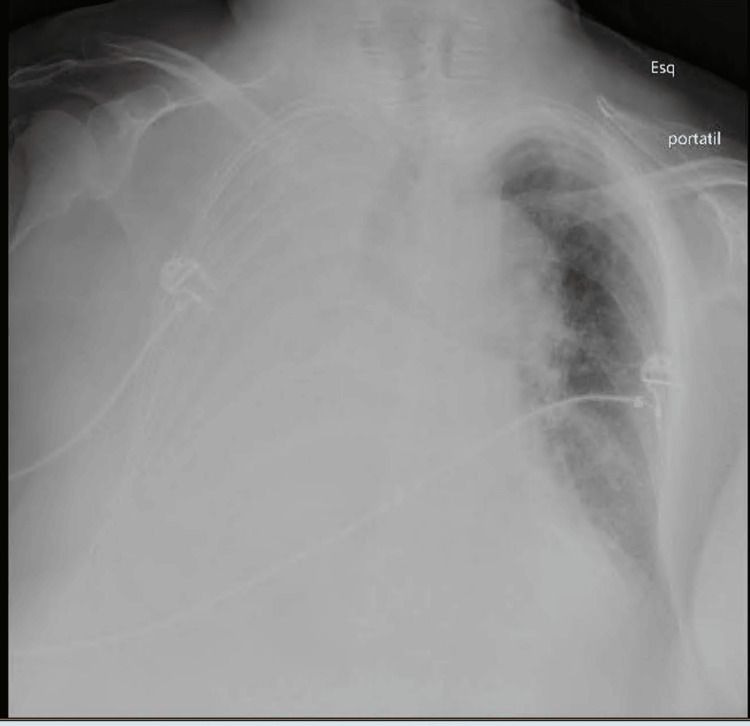
Anteroposterior chest X-ray showing extensive right pleural effusion.

**Figure 2:**
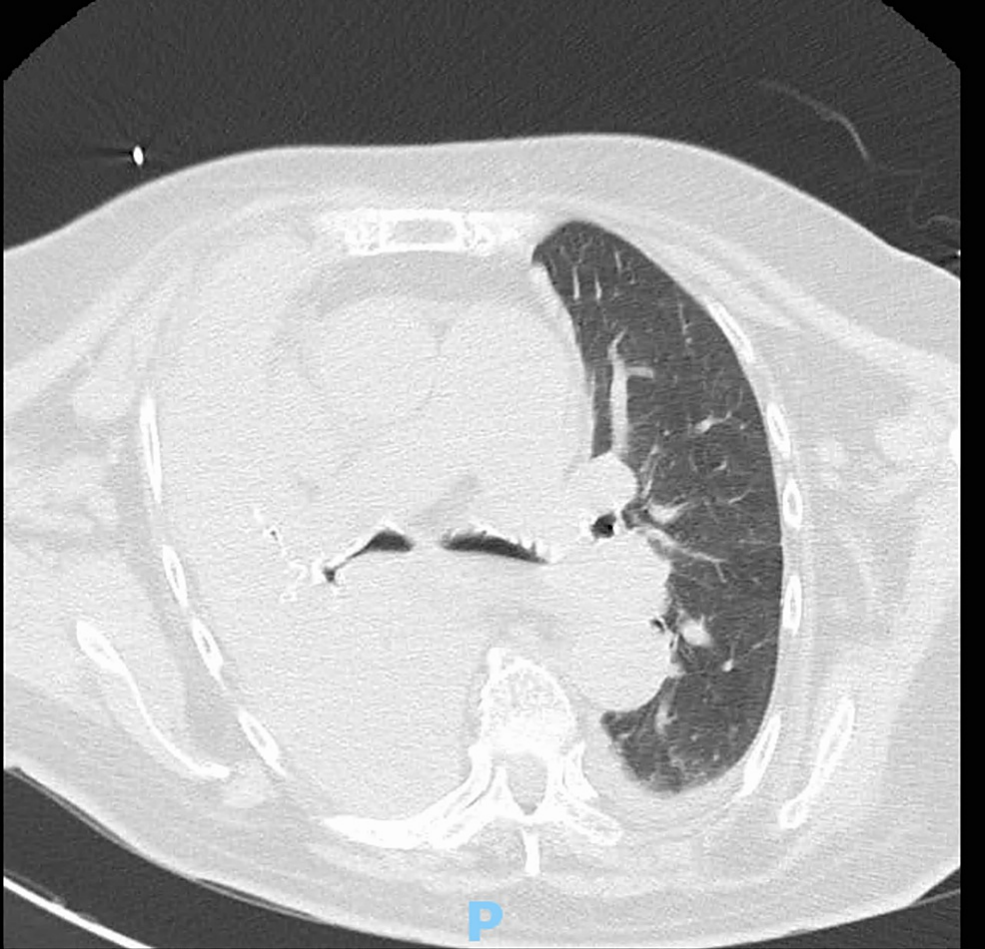
A CT with angiography of the chest showing significant right pleural effusion with associated right-lung collapse.

Upon admission, she was lethargic but arousable, tachypneic with supplemental oxygen of 10 L per minute, normotensive, and tachycardic. Pulmonary auscultation revealed globally decreased breath sounds. Arterial blood gas analysis with supplementary oxygen exhibited respiratory acidemia (pH of 7.088, unmeasurable partial pressure of carbon dioxide (pCO2), partial pressure of oxygen (pO2) of 76.5 mmHg, saturation of 93.5%, normal lactate, and bicarbonate). No laboratory alterations were noted. A chest X-ray revealed a pleural effusion occupying the entire right lung (Figure 1). The patient underwent a CT with angiography, demonstrating bilateral pleural effusion, moderate on the right with associated right-lung collapse, and signs of acute central pulmonary embolism of the left basal pyramid segmental branches (Figure 2).

**Figure 3:**
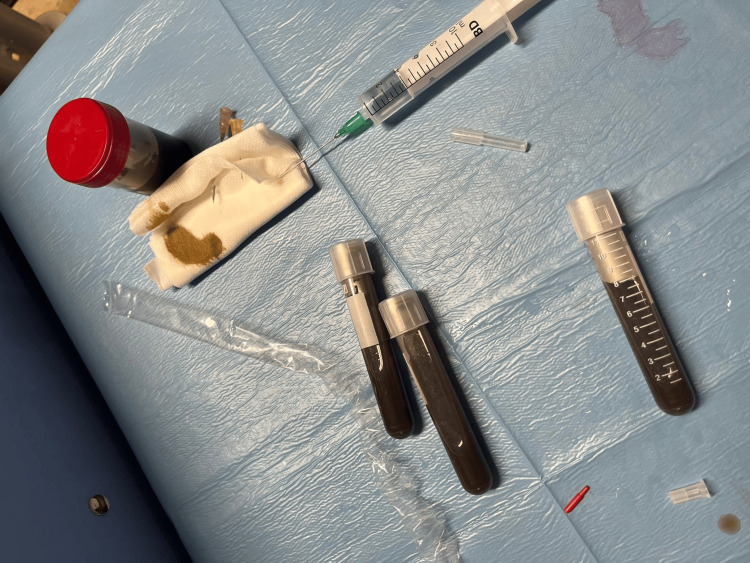
Bilious pleural fluid thoracocentesis sample.

**Figure 4:**
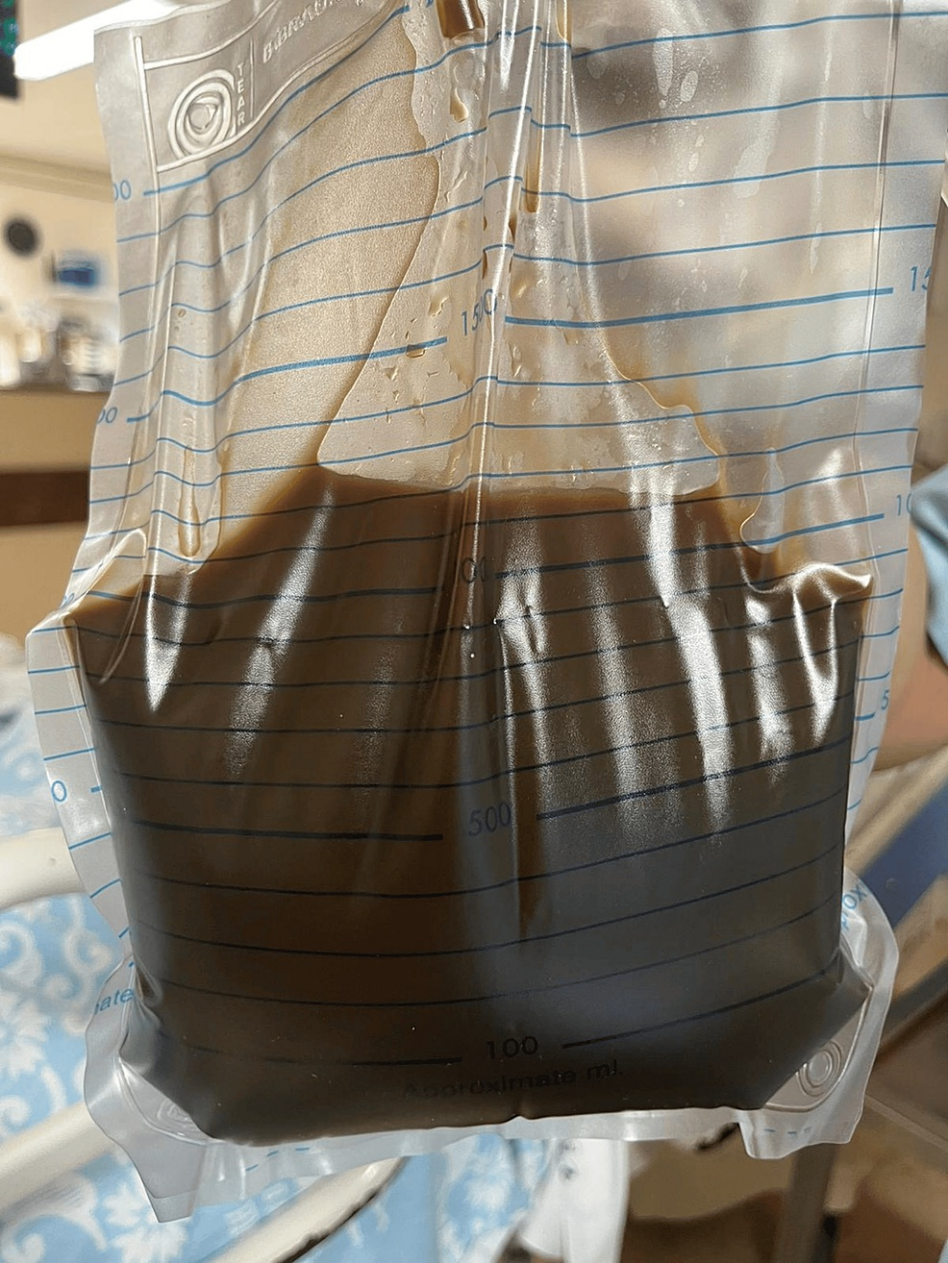
Bilious pleural fluid drainage.

A thoracentesis was performed, revealing a dark greenish fluid (Figures 3,4). An effusion analysis confirmed the presence of an exudate according to the Light criteria (Table 1).

**Table 1:** Thoracocentesis pleural fluid analysis.

Pleural fluid	Patient value	Reference range
pH	8.5	7.6-7.64
Leucocytes (cells/mm^3^)	<30 cells/mm^3 ^(predominantly mononuclear cells with polyhedral crystals and granules)	<1000
Glucose (mg/dL)	21	70-100
Pleural proteins/serum proteins (g/dL)	16.6/5.7	1-2/6-8.3
Pleural lactate dehydrogenase/serum lactate dehydrogenase (IU/L)	4500/165	50% of serums/140-280
Adenosine deaminase (ADA, IU/L)	173.8	0-30
Ratio of pleural total bilirubin (mg/dL) and serum total bilirubin (mg/dL)	6.1/0.5=12.2	<1
Serum total bilirubin (mg/dL)	0.5	<1.2

A comparison of pleural total bilirubin to plasma total bilirubin confirmed the suspicion of biliothorax.

Because of biliothorax without known biliary pathology or surgical history, an abdominal-pelvic CT was requested, revealing no abnormal findings, namely, biliary alterations or fistula.

Because of refractory shock and deterioration of clinical status, the patient was admitted to the intensive care unit (ICU). Pleural fluid cultures were negative, but histopathology revealed positive results for neoplastic cells: lung adenocarcinoma (TTF-1 positive). Red blood cells are often found in pleural fluid because of malignancy, these cells may suffer apoptosis before their 120-day lifespan, leading to a hemolytic process with the production of bilirubin. Therefore, a case of biliothorax in a likely hemolytic context of paraneoplastic pleural effusion was proposed.

Unfortunately, the patient passed away in the ICU.

## Discussion

Most cases of biliothorax are associated with malignant or benign biliary obstruction, pleurobiliary fistulas after hepatobiliary procedures, and hepatic or subphrenic abscesses [2,3,6,7]. The diagnosis of biliothorax requires a high index of clinical suspicion and pleural fluid analysis [4]. A ratio of total pleural fluid bilirubin to total serum bilirubin greater than one confirms the diagnosis [2,3,5].

A delay in the diagnosis and management of biliothorax can be life-threatening, leading to serious pleural complications and sepsis [1]. Bile acid is a potent irritant, leading to a significant inflammatory response, creating an environment for infection in the pleural space that may result in empyema [2]. Early recognition and complete drainage are important for preventing these complications [3,4]. Treatment involves the drainage of pleural effusion with a chest tube and biliary stenting if surgical etiology is identified to reduce ductal pressure [2].

The presented case describes a patient with an acute onset of dyspnea and an extensive pleural effusion that, after thoracocentesis, was revealed to be bile in the pleural cavity. Biliothorax is a very rare clinical event and even rarer in a patient with no known biliary pathology or surgical history. There is very little literature on biliothorax cases, and an exhaustive review retrieved no cases of hemolytic biliothorax, highlighting the importance of the case described.

## Conclusions

Bile in the pleural cavity is a rare clinical event, and there is very little literature on biliothorax cases, especially of nonsurgical origin. The presented case illustrates a woman with pulmonary adenocarcinoma, experiencing biliothorax as a complication. A high level of suspicion is necessary for a prompt diagnosis when faced with an effusion liquid of a dark greenish color, and fast drainage is essential to prevent life-threatening complications.
